# PURPOSE IN LIFE ACROSS THE COVID-19 PANDEMIC: THE EFFECTS OF COGNITIVE ABILITY, AGE, AND GENDER

**DOI:** 10.1093/geroni/igad104.1026

**Published:** 2023-12-21

**Authors:** Niccole Nelson, Cindy Bergeman

**Affiliations:** Colorado State University, Fort Collins, Colorado, United States; University of Notre Dame, Notre Dame, Indiana, United States

## Abstract

In March of 2020, the World Health Organization declared COVID-19 a pandemic. Almost immediately, 95% of the US population was under stay-at-home orders, resulting in widespread uncertainty about the future, low community engagement, and social isolation. These effects may have been detrimental to maintaining a sense of purpose in life across this period, which has been linked to several health and wellness factors, including cognitive ability. Although previous work has indicated that higher cognitive ability predicts a stronger sense of purpose in life, the relationship between cognitive ability and changes in purpose across this universal, chronic stressor is unknown. The present study, therefore, examined the effects of cognitive ability, age, and gender on trajectories of purpose in life across the COVID-19 pandemic. Participants included 206 individuals (Age Range=45-88, M=69.04, SD=9.29) who participated in a cognitive assessment before the pandemic, as well as the multi-wave Notre Dame Study of Health & Well-being-COVID Study (NDHWB-CS) from September 2020-February 2022. Two-level, multilevel modeling tested the effects of total, fluid, and crystalized cognitive abilities, as well as age and gender, on trajectories of purpose in life. Results indicated that greater total and fluid cognitive abilities were associated with higher initial levels of purpose in life. Furthermore, although crystalized, fluid, and total cognitive abilities were each associated with change in purpose across the pandemic, there were age and gender differences in these effects. Theoretical and practical implications regarding the relationship between cognitive ability and sense of purpose in the context of stress will be discussed.

